# Monitoring geological storage of CO_2_ using a new rock physics model

**DOI:** 10.1038/s41598-021-04400-7

**Published:** 2022-01-07

**Authors:** Manzar Fawad, Nazmul Haque Mondol

**Affiliations:** 1 Department of Geosciences, University of Oslo, Oslo, Norway; 2 Norwegian Geotechnical Institute (NGI), Oslo, Norway

**Keywords:** Carbon capture and storage, Geophysics

## Abstract

To mitigate the global warming crisis, one of the effective ways is to capture CO_2_ at an emitting source and inject it underground in saline aquifers, depleted oil and gas reservoirs, or in coal beds. This process is known as carbon capture and storage (CCS). With CCS, CO_2_ is considered a waste product that has to be disposed of properly, like sewage and other pollutants. While and after CO_2_ injection, monitoring of the CO_2_ storage site is necessary to observe CO_2_ plume movement and detect potential leakage. For CO_2_ monitoring, various physical property changes are employed to delineate the plume area and migration pathways with their pros and cons. We introduce a new rock physics model to facilitate the time-lapse estimation of CO_2_ saturation and possible pressure changes within a CO_2_ storage reservoir based on physical properties obtained from the prestack seismic inversion. We demonstrate that the CO_2_ plume delineation, saturation, and pressure changes estimations are possible using a combination of Acoustic Impedance (AI) and P- to S-wave velocity ratio (Vp/Vs) inverted from time-lapse or four-dimensional (4D) seismic. We assumed a scenario over a period of 40 years comprising an initial 25 year injection period. Our results show that monitoring the CO_2_ plume in terms of extent and saturation can be carried out using our rock physics-derived method. The suggested method, without going into the elastic moduli level, handles the elastic property cubes, which are commonly obtained from the prestack seismic inversion. Pressure changes quantification is also possible within un-cemented sands; however, the stress/cementation coefficient in our proposed model needs further study to relate that with effective stress in various types of sandstones. The three-dimensional (3D) seismic usually covers the area from the reservoir's base to the surface making it possible to detect the CO_2_ plume's lateral and vertical migration. However, the comparatively low resolution of seismic, the inversion uncertainties, lateral mineral, and shale property variations are some limitations, which warrant consideration. This method can also be applied for the exploration and monitoring of hydrocarbon production.

## Introduction

Subsurface CO_2_ storage is not a new concept. For decades, the oil and gas industry has been re-injecting the CO_2_ produced along with the hydrocarbon gases^1,2^. CO_2_ injection has also been used for enhanced oil recovery^3,4^. Carbon capture and storage (CCS) has the potential to significantly reduce CO_2_ build-up in the atmosphere from fossil fuel use; however, large-scale subsurface CO_2_ storage still may pose different technical and social challenges^5^.

Buoyancy trapping is the key process for CO_2_ storage during the injection and early stage of storage^5^. Therefore, the CO_2_ is injected at the base of the reservoir, and the plume migrates laterally within the most permeable beds until it finds a vertical passage (fault or fracture) to move upwards and accumulate below the base of the caprock. The plume behavior is a function of the horizontal and vertical heterogeneities within the reservoir. The thin clay and silt layers or carbonate laminations may facilitate lateral distribution of CO_2_ in the storage reservoir. For example, in the Sleipner CCS project, the four-dimentional (4D) or time-lapse seismic enables one to trace the migration path and subsequent accumulation of the CO_2_ plume^6^. The other CO_2_ trapping mechanisms are residual gas trapping, solubility trapping, and mineral trapping. The time-lapse or 4D seismic is carried out to monitor the CO_2_ plume migration within the storage reservoir (for example, in a saline aquifer), and to identify a possible vertical CO_2_ leakage into the shallower strata or surface.

There are several methods in use for seismic fluid prediction^7^. Many provide qualitative hydrocarbon indication, whereas few techniques are quantitative. The qualitative methods comprise Amplitude-Variation-with-Offset (AVO) analysis^8–11^, AVO cross plotting^12,13^, Lambda-Mu-Rho (LMR)^14^, Extended Elastic Impedance (EEI)^15^, and Curved Pseudo Elastic Impedance (CPEI)^16,17^. The examples of quantitative methods are Acoustic Impedance versus P- to S-wave velocity ratio (AI-versus-Vp/Vs) rock physics template^18–20^, Multi-Attribute Rotation Scheme (MARS)^21^, Inverse Rock Physics Modelling (IRPM)^22,23^, and technique to discriminate saturation and pressure from 4D seismic using near and far offset stacks^24^.

A practical approach suggested for fluid saturation discrimination^25^ using seismic data employed a method similar to LMR^14^. Lame parameters were calculated; however, the fluid saturation was suggested to be estimated on a ρ/μ versus λ/μ plane as opposed to the LMR method where a λρ versus μρ was used to differentiate various facies (ρ is bulk density, λ is incompressibility, and μ is shear modulus). Two-dimentional permeability modelling^26^ of CO_2_ saturation, distribution, and seismic response showed CO_2_ trapping, and the P-wave velocity (Vp) and water saturation (Sw) relationship were mostly a function of the Dykstra-Parson^27^ coefficients. Executing a workflow for forward modeling^28^ of time-lapse seismic data indicated that a high signal-to-noise ratio was needed to detect the CO_2_ leakage at the model site. Both^26,28^ the studies used Gassmann equations^29^ for fluid substitution. Another three-dimentional (3D) modelling study^30^ related AI changes with the water saturation (Sw), and quantitatively demonstrated that seismic amplitudes can be more precise than seismic impedances for quantifying Sw changes with 4D seismic data.

A seismic profile can be defined as an array of processed seismic traces. Each trace represents the convolution of a source wavelet with an input reflectivity sequence where each reflectivity spike depicts the contrast in acoustic impedance (AI = P-wave velocity × Bulk Density) across a geological interface. A seismic inversion is carried out to convert the interface property (reflectivity) to a physical rock property such as AI^31,32^. With the advent of AVO/prestack inversion, it became possible to obtain the shear wave (Vs) information also, usually in the form of shear impedance (SI) from the AVO far-offset data. Various forms of Fatti's equation^33^ are used for AVO inversion; one of that is^34^:

1 $${R}_{P}\left(\theta \right)\approx \left(1+{tan}^{2}\theta \right)\frac{\Delta AI}{2AI}-8{\left(\frac{{V}_{S}}{{V}_{P}}\right)}^{2}{sin}^{2}\theta \frac{\Delta SI}{2SI}$$

where *R*_*P*_(θ) is the P-wave reflectivity at an angle θ, this angle is the average of incidence and transmission angles, Vp is P-wave velocity, Vs is S-wave velocity, ΔAI/2AI and ΔSI/2SI are acoustic impedance and shear impedance reflectivities, respectively.

Rock physics models represent the link between the reservoir properties (e.g., porosity, clay content, sorting, lithology, saturation) and seismic-derived elastic properties (e.g., AI, SI, or Vp/Vs ratio). One of the existing models comprised a hybrid modeling approach^19^ using the AI versus Vp/Vs RPT applied specifically to sandstones employing a physical-contact theory, i.e., the Hertz-Mindlin model^35^ combined with theoretical elastic bounds, e.g., the Hashin–Shtrikman bounds^36^ simulating the porosity reduction trend associated with depositional sorting and diagenesis. For soft shales, the seismic properties were estimated as a function of pore shape. Gassmann fluid substitution^29^ was carried out to estimate the effect of varying gas versus water saturation in the sand layers, whereas Backus average^37^ was used to predict the effective seismic properties for changing net-to-gross (N/G ratios)^19^. However, it has been demonstrated^22^ that even with the standard rock physics template (RPT) of AI versus Vp/Vs^18–20^, it is difficult to know whether the model is adequately calibrated to the data or how it can be interpreted. Furthermore, there are nonunique solutions resulting in various combinations of porosity, lithology, and fluid saturations that have the same Vp/Vs ratio and AI, using the same rock physics model^22^.

In this study, we introduce a new interactive rock physics model that directly relates AI with the Vp/Vs ratio for predicting fluid saturation (S_fl_). The model can be calibrated with the well-log data interactively, without using the Hertz-Mindlin model^35^, Hashin–Shtrikman bounds^36^, or Gassmann fluid substitution^29^. The suggested model is nonlinear similar to CPEI^16,17^, but with physical meanings and flexibility that can readily be applied to the seismic-derived AI and Vp/Vs cubes to estimate S_fl_. We came up with a similar equation in a previous publication^38^ to calculate shale volume (Vsh) based on the AI, Vp/Vs ratio domain.

Following is the proposed model to estimate the target fluid saturation (S_fl_) in fraction using the AI and Vp/Vs ratio data obtained by AVO inversion:

2 $${S}_{fl}=\frac{\left\{{\rho }_{ma}+\left[1-{\left(\frac{{V}_{S}}{{V}_{P}G\propto }\right)}^\frac{1}{n}\right]\left({\rho }_{w}-{\rho }_{ma}\right) - AI \left[\frac{1}{{V}_{Pma}}+\left(1-{\left(\frac{{V}_{S}}{{V}_{P}G\propto }\right)}^\frac{1}{n}\right) \left(\frac{1}{{V}_{Pw}} - \frac{1}{{V}_{Pma}}\right)\right]\right\}}{\left\{\left[1-{\left(\frac{{V}_{S}}{{V}_{P}G\propto }\right)}^\frac{1}{n}\right] \left[AI\left(\frac{1}{{V}_{Pfl}} - \frac{1}{{V}_{Pw}}\right)-\left({\rho }_{fl}-{\rho }_{w}\right)\right]\right\}}$$

where V_Pma_ and V_Pw_ are the P-wave velocities of the mineral matrix, and brine respectively, V_Pfl_ is the apparent P-wave velocity of the target fluid, ρ_ma_ is the density of mineral grains, ρ_fl_ is the apparent density of the target fluid, ρ_w_ is the density of brine, AI is acoustic impedance, G is the mineralogy/shaliness coefficient, α is Vs/Vp ratio of the mineral/rock matrix, and n is the stress/cementation coefficient. The water saturation (S_w_) can be calculated subsequently (S_w_ = 1 − S_fl_).

**Figure 1:**
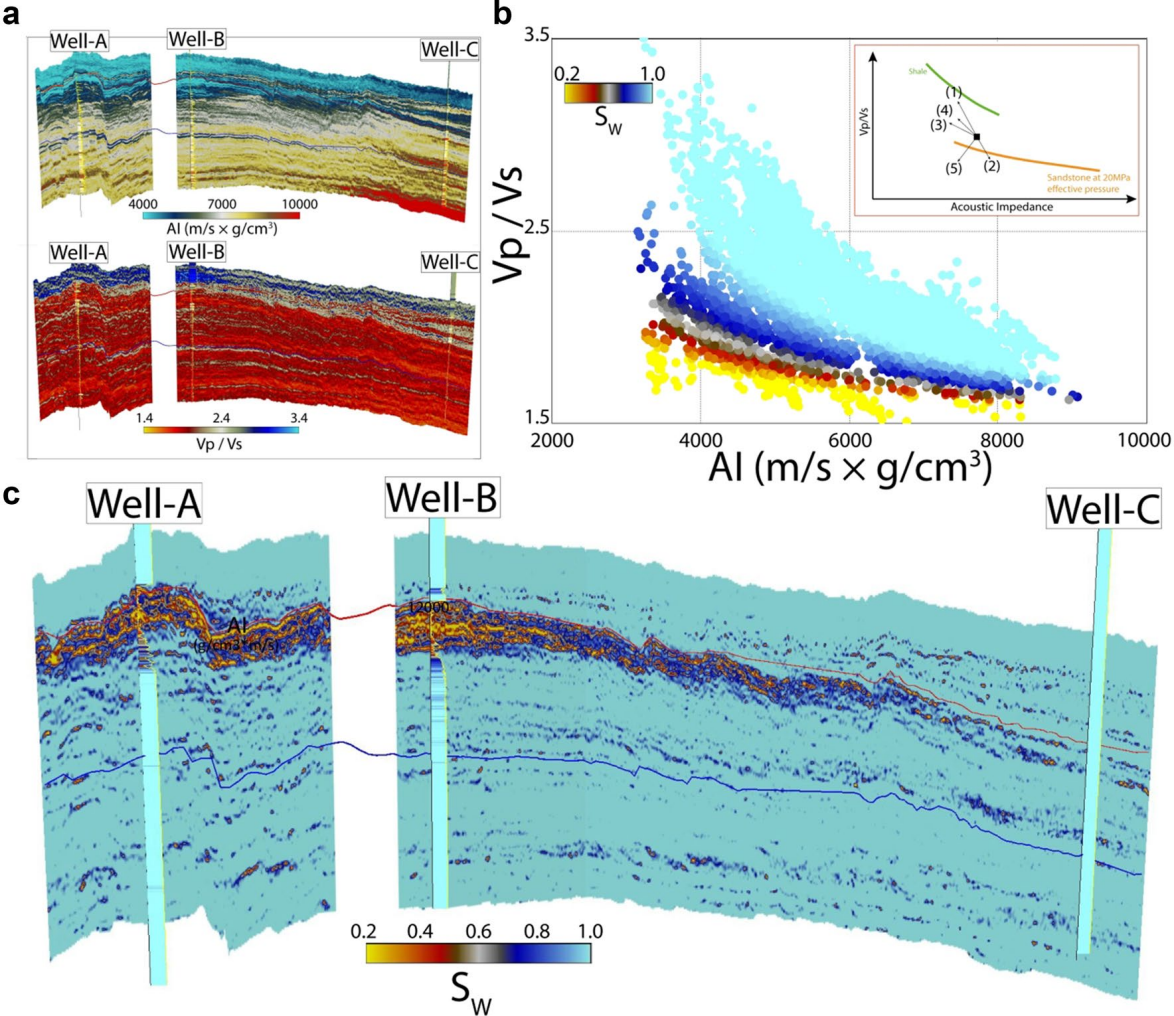
An example of a fluid response in a hydrocarbon field on the Norwegian Continental Shelf, (**a**) AI and Vp/Vs ratio profiles obtained from a seismic inversion with hydrocarbon-bearing wells (Well-A, Well-B), and a dry well (Well-C), (**b**) Data along the seismic lines plotted on the AI-Vp/Vs plane show that the fluid effect can be isolated and quantified using our proposed rock physics model, (**c**) the resulting fluid saturation profile indicating the hydrocarbon anomaly and it's extent. The inset in (**b**) does also show how the brine saturated sandstone will plot as the (1) shale content increase, (2) the amount of cement increase, (3) the porosity in the sandstone increase, (4) the effective stress in the formation decrease and (5) the saturation of gas increase within the sandstone^18^.

As mentioned previously, the AI and Vp/Vs ratio are obtained by inverting seismic data (Fig. 1a). AI increases, and Vp/Vs ratio decreases typically with increasing burial depth due to a decrease in porosity. If a low-density fluid (hydrocarbon or CO_2_) replaces the in-situ brine, a reduction both in AI and Vp/Vs values is expected depending upon the substituted fluid's density. We came up with Eq. (2) that relates AI with Vp/Vs ratio to isolate the target fluid saturation from the brine saturated sandstone compaction trend on the AI versus Vp/Vs ratio plane (Fig. 1b, c). One can calibrate the model using nearby well data (Well-A in this case, see “Methods” section).

This technique will help to monitor a CO_2_ plume in the subsurface for lateral and vertical migration. For saturation estimation of a particular CO_2_ phase (e.g., gas, supercritical or liquid), the input V_Pfl_ (apparent P-wave velocity of the target fluid) and ρ_fl_ (apparent density of the target fluid) can be defined accordingly. The proposed method will be useful for reliable control on the CO_2_ injection and sequestration processes. Other uses could be oil and gas production monitoring and hydrocarbon exploration.

**Figure 2:**
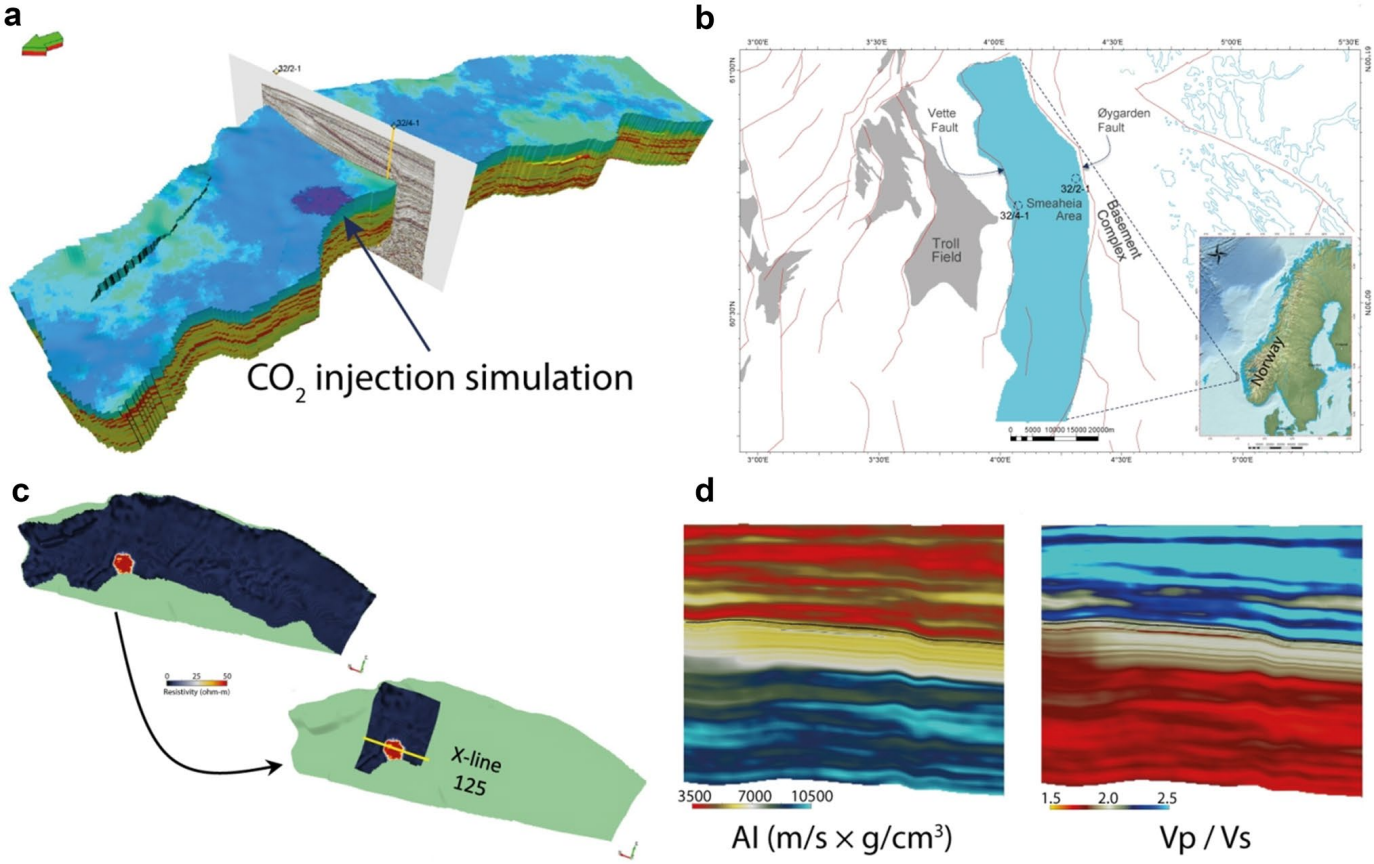
(**a**) The original Northern Light project^41^ simulation modelling grid, (**b**) location of the modelled grid area (light blue) in the northern North Sea, maps modified from the Norwegian Petroleum Directorate (NPD) data^43^, c) example of a property grid carved out to a seismic formatted cube covering only the injection and storage area, (**d**) AI and Vp/Vs ratio profiles along crossline 125 shown in (**c**), the example here is of the year 2050, the effect of injected CO_2_ on both AI and Vp/Vs ratio is very subtle (Figure modified after^39^).

**Figure 3:**
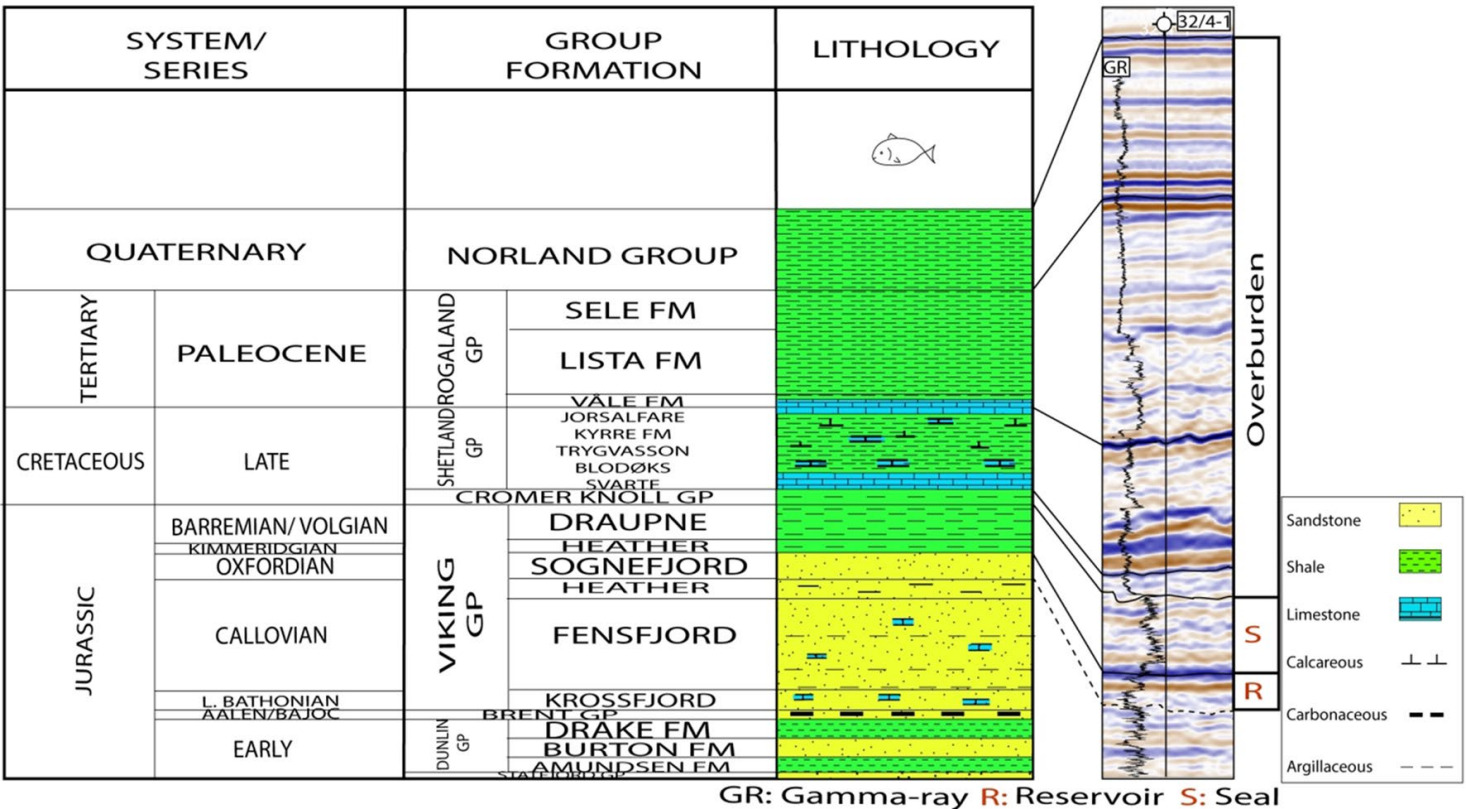
A generalized Jurassic to Quaternary stratigraphic succession in the study area (modified from^38,44^). The base Sognefjord Fm contact with Heather Fm is not so obvious on seismic; therefore, it is shown as a dotted line.

Similar to our previous study^39^, we used the synthetic elastic property data from the Norwegian Geotechnical Institute (NGI). NGI generated Vp, Bulk Density, and Resistivity^40^ properties using grids from a reservoir model by the Northern Light project^41^ (Fig. 2a). Additionally, we calculated the Vs data to generate the Vp/Vs ratio cubes (see details in the “Methods” section). The reservoir model was a simulation of one of the potential CO_2_ storage sites in the northern North Sea called Smeaheia (Fig. 2b). The Smeaheia area is bounded by a fault array separating the Troll oil and gas field in the west and the Basement Complex in the east^38^. The primary CO_2_ storage reservoir in the Smeaheia area is Sognefjord Formation (Upper Jurassic) sandstone, capped by the Draupne and Heather Formation (Upper Jurassic) shales^38,42^ (Fig. 3). The amount of CO_2_ to be stored was 1.3 Mt/year employing an injection period of 25 years with an injection rate of 200 tons/hr. We sliced out the AI and Vp/Vs ratio cubes covering only the injection and storage area to reduce computation time and converted the cubes to a depth-domain seismic format with inline and crossline profiles (Fig. 2c). We assumed that the AI and the Vp/Vs cubes were the actual values obtained from the seismic inversion (Fig. 2d).

We assumed a monitoring scenario over 40 years, with injection starting in 2020 for 25 years, keeping an assumption that the time-lapsed seismic surveys were acquired every 10 years. This study also has implications for hydrocarbon exploration and monitoring of oil and gas production. The anisotropy in physical properties, CO_2_ dissolution, and chemical reaction with rock grains and their effect on the AI and Vp/Vs ratio are not taken into account.

## Results and discussion

**Figure 4:**
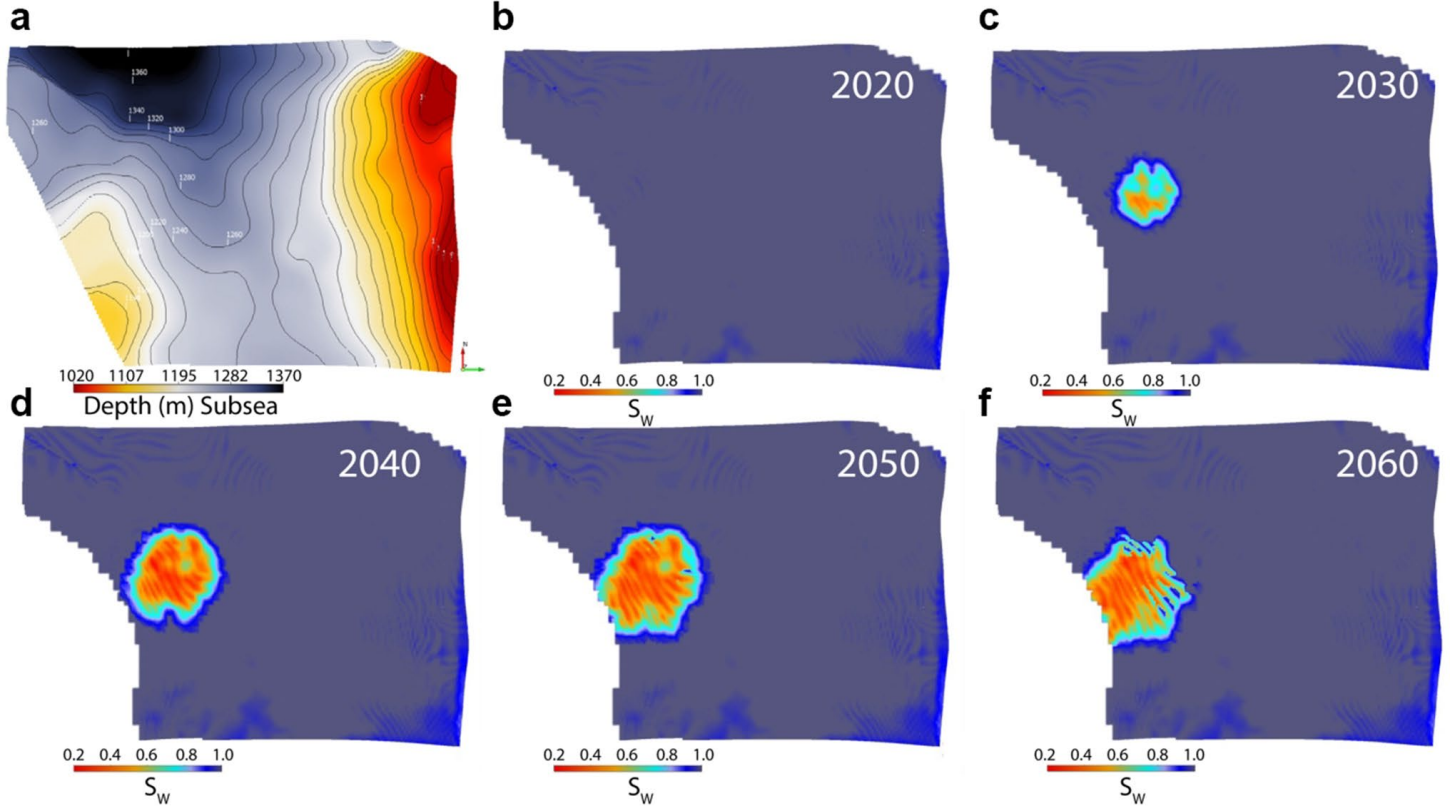
The top Sognefjord Formation reservoir depth surface (**a**) draped on saturation cubes in years (**b**) 2020, (**c**) 2030, (**d**) 2040, (**e**) 2050, and (**f**) 2060. The CO_2_ plume moves up-dip over time towards the southwest.

We demonstrate a scenario where we have time-lapsed/4D seismic data from 2020 before injection to the year 2060. The top of the Sognefjord Formation reservoir lies between 1020 and 1370 m below mean sea level (Fig. 4a). The reservoir is brine saturated before CO_2_ injection in 2020 (Fig. 4b). Both the reservoir AI and Vp/Vs ratio supposedly obtained from prestack inversion decreases where the CO_2_ plume replaces the in-situ brine. Therefore, the estimated saturations from AI and Vp/Vs ratio clearly define the plume boundaries and reservoir inhomogeneity (Fig. 4c–f). We can also see the plume boundary systematically increasing with the passage of years and moving towards the southwest in the up-dip direction. The injection stopped in 2045, therefore a water breach within the plume along the northeastern boundary is apparent as the plume migrates southwestwards in the panel showing the year 2060 (Fig. 4f).

**Figure 5:**
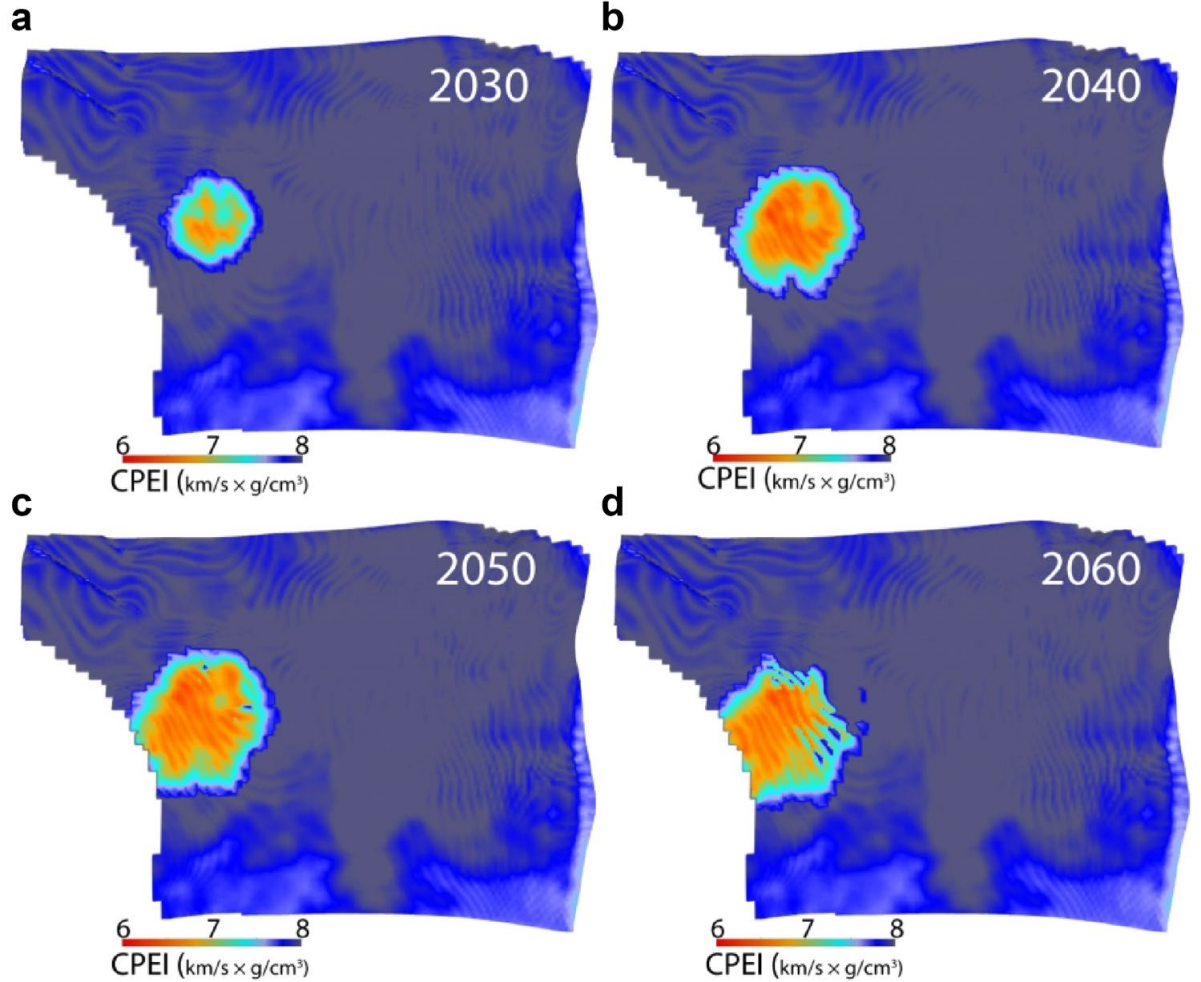
The top Sognefjord Formation reservoir depth surface draped on Curved Pseudo Elastic Impedance (CPEI)^17^ attribute cubes in years (**a**) 2030, (**b**) 2040, (**c**) 2050, and (**d**) 2060. The CPEI anomalies effectively demarcate the CO_2_ plume in the respective year of survey; however, it is difficult to relate the CPEI value with a certain CO_2_ saturation.

For comparison, we used the Curved Pseudo Elastic Impedance (CPEI)^17^ attribute to observe the CO_2_ plume effect (Fig. 5). CPEI is a fixed function with coefficients controlling the wet-rock trend and grain mineralogy. Qualitatively, the CPEI fluid-related anomalies are almost identical to that of estimated using Eq. (2) (Fig. 4) for the respective survey years, as both the functions are essentially non-linear. In theory, the CPEI values less than 6.9 (km/s × g/cm^3^), here denoted by hot colours, should represent the fluid softening due to CO_2_ replacing the in-situ brine^16,17^. However, it can be noticed that the CPEI anomaly values extend above 6.9 (km/s × g/cm^3^), making it difficult to relate it with actual CO_2_ saturation within the reservoir.

### Discrimination between pressure and fluid saturation affects

**Figure 6:**
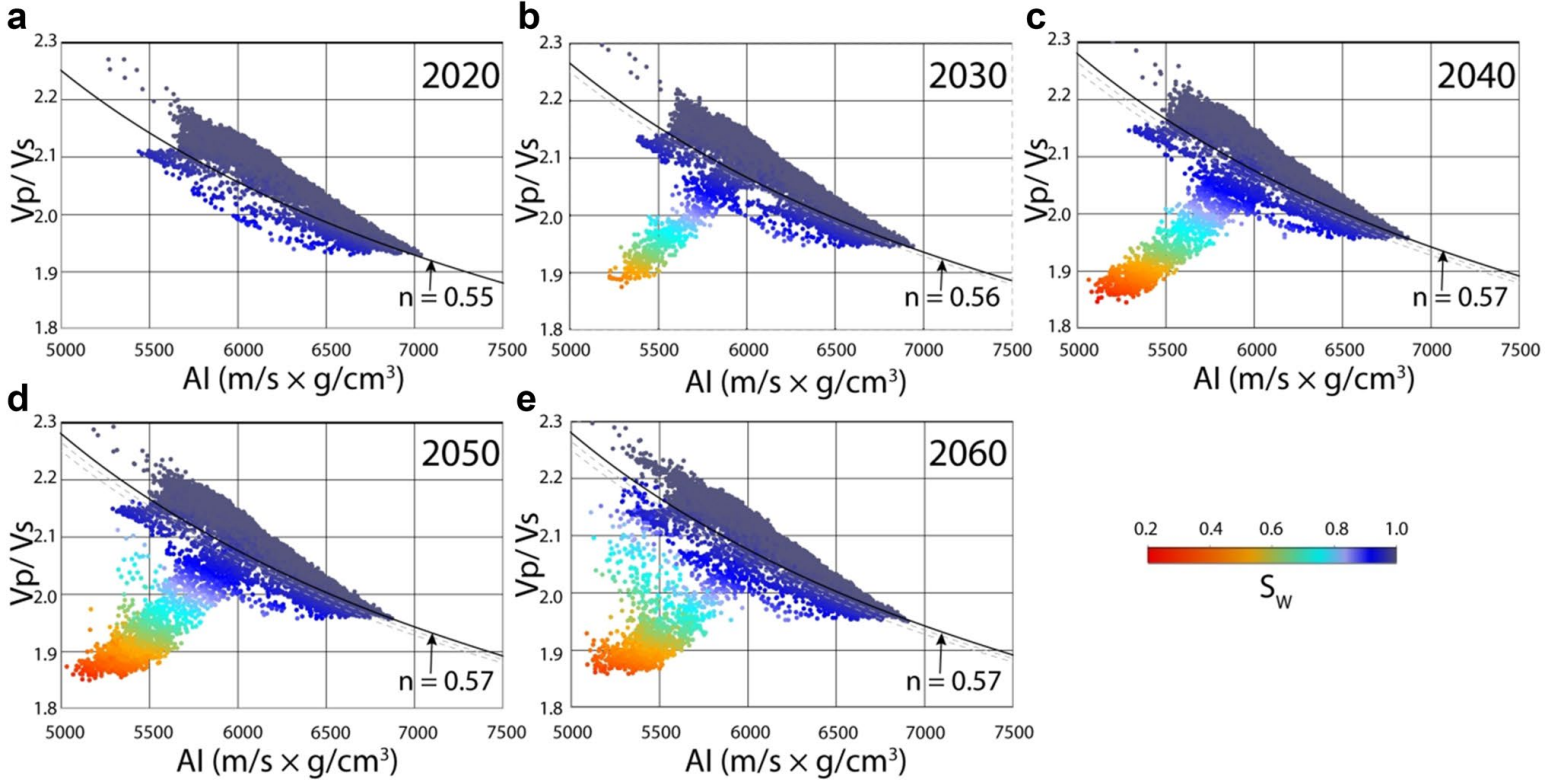
Data points sampled at regular intervals on the top Sognefjord Formation sandstone surface are displayed on the AI versus Vp/Vs ratio plane colour-coded by S_w_ for years (**a**) 2020, (**b**) 2030, (**c**) 2040, (**d**) 2050, and (**e**) 2060. The position of the brine-saturated sandstone line with corresponding 'n' values is also shown in each panel.

On the AI versus Vp/Vs crossplot, there is a systematic decrease in water saturation within reach of the CO_2_ plume from 2020 to 2060 (Fig. 6). The CO_2_ injection started in 2020 and was completed in 2045. In the panels representing the year 2050, the gas saturated points show a little scatter that increases in 2060. This point scatter could be due to the diffusion and up-dip migration of gas.

With the increase in time from 2020 to 2040, there is a subtle shift in the brine-sand trend (Fig. 6a–c) in the direction '4' shown in the inset of Fig. 1b. We calibrated the brine-sand trend for saturation calculations by changing the value of stress/cement coefficient 'n'. This change in 'n' values is a good indication of reducing effective stress due to the increase in pore pressure (approximately 10 Bar/1 MPa). The brine-saturated sand trend stays roughly the same in the panel covering the end of injection year, i.e., 2045 (Fig. 6d), and the subsequent survey in 2060 (Fig. 6e). One should bear in mind that the Sognefjord Formation sandstone reservoir is predominantly un-cemented^38^. We cannot expect a similar change of brine trend with a change in effective stress within deeper quartz cemented sandstones. Relating the change in 'n' values with the effective stress in various un-cemented sands needs further studies.

### Advantages of our suggested rock physics model

**Figure 7:**
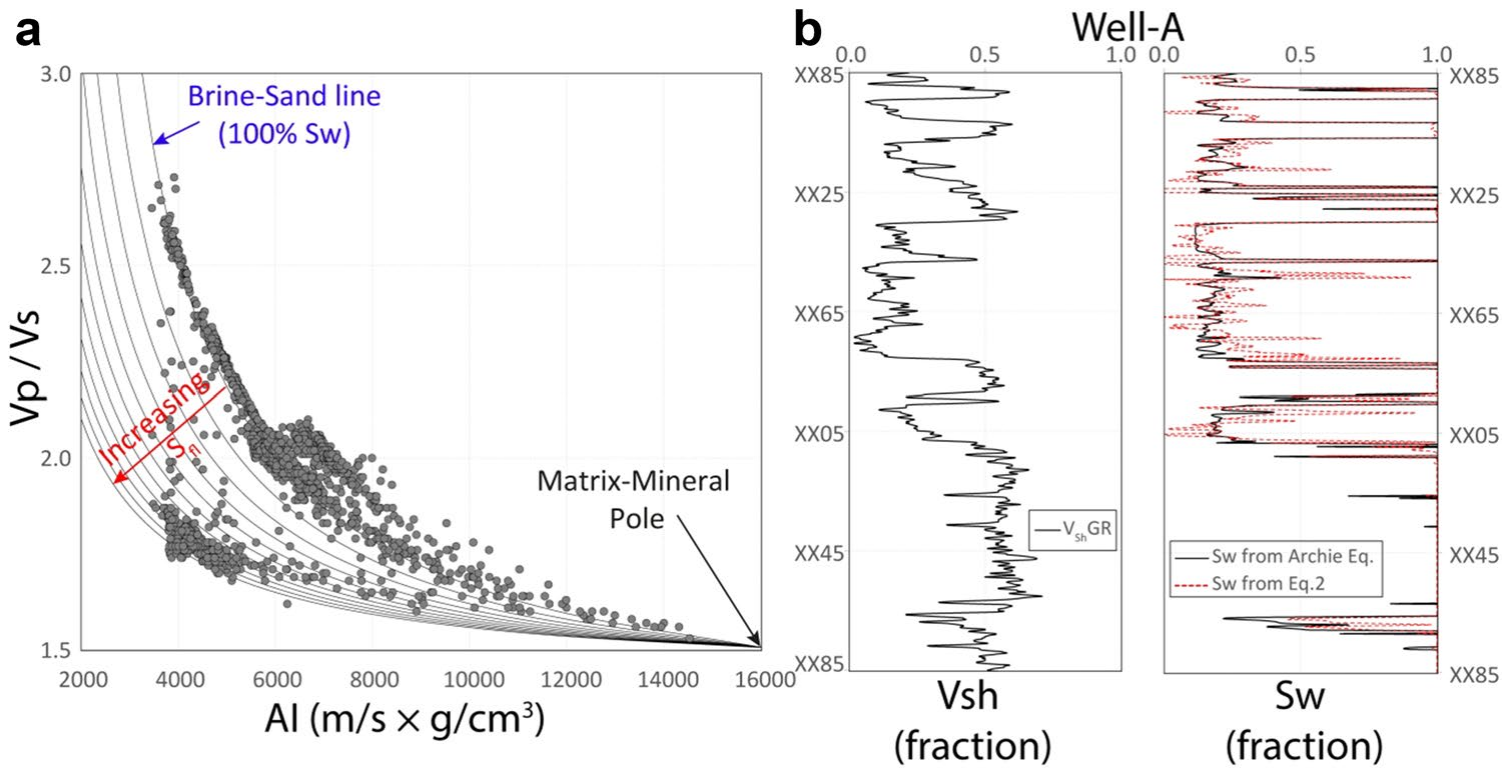
Method of calibrating the rock physics model. (**a**) Aligning the brine-saturated sandstone trend in the data with the reference 100% S_w_ line onto the Acoustic impedance versus Vp/Vs ratio plane by iterating the 'G' and 'n' values. (**b**) Calibrating S_w_ by iterating the apparent velocity and density of the hydrocarbon until the S_w_ curve is obtained from Eq. (2) correlates with the Archie S_w_^49^.

In the traditional AI-Vp/Vs rock physics template^18,19^, the dry sandstone is modeled by combining Hertz-Mindlin contact theory^35^ and Hashin-Shtrikman^36^ interpolation, and finally, Gassmann fluid substitution^29^ is performed to estimate the effect of varying fluid saturation in the sand layers. The modelling typically starts from the high-porosity end member interpolated to zero porosity matrix mineral point employing equations that use the rock bulk (*K*) and shear (*μ*) moduli as input. The model we suggested (Eq. 2) does not require computations at the elastic moduli level. The matrix pole/point is defined on the AI versus Vp/Vs plane on the basis of coefficient α that is Vs/Vp ratio of the mineral/rock matrix (Fig. 7). While keeping the matrix point at the same position, the gradient of the line interpolating between the matrix point with the high-porosity end member can be changed using the coefficient ‘n’. This interpolation defines the brine-sand (100% Sw) line that can be adjusted to calibrate with the stress or cementation condition of the target layer. Changing the shale/mineralogy coefficient ‘G’ results in a static vertical shift of the brine-sand line that helps adjust with the N/G ratio of the target layer data. The saturation contours adjust themselves with respect to the brine-line according to the given apparent P-wave velocity and density of the target fluid (V_Pfl_ and ρ_fl_, respectively). This procedure does not require Gassmannn substitution^29^ as one needs in the traditional AI-Vp/Vs rock physics template. Also, the model works for both un-cemented and cemented sandstones. In the case of Extended Elastic Impedance (EEI)^15^, the calculated properties (for instance, Sw) appear linear on the AI-Vp/Vs ratio plane; however, the actual sandstone exhibits a non-linear curvature^16^. This nonlinearity is captured by our model, same as the curved pseudo-elastic impedance (CPEI)^16,17^ (Fig. 5); however, our suggested model is quantitative and, as discussed above, flexible in terms of grain mineralogy and fluid density. The LambdaRho-MuRho^14^ calculations to differentiate lithology and fluid content introduce error and bias because of squaring the impedances^18^. The equation we present does not contain any squared factors, thus preventing additional errors.

For subsurface storage, CO_2_ is injected in the supercritical phase to a depth where the temperature and pressure keep the gas in the same phase. This approach maximizes the use of available storage volume in the pore spaces within a reservoir. Therefore, the optimum depth for storage is from 1 to 3 km depth^5^. The quartz cementation approximately starts below 2000 m from the seafloor in the North Sea, where the temperature becomes more or less 70 °C. We demonstrated that there is a possibility of quantifying the change in pressure within the un-cemented reservoir sands; therefore, using our suggested model will be helpful in that case. In both un-cemented and cemented sandstone reservoirs, if the supercritical CO_2_ plume converts to gas at some point in time due to a decrease in pore pressure, the subsequent time-lapse S_w_ calculations using our model will yield a value less than zero indicating a pressure drop.

### Limitations and pitfalls

This method can be applied only in siliciclastics as the carbonates exhibit a different Vp to Vs relationship. There is a difference in resolution between the wireline log data and seismic; therefore, calibrating the model using wireline logs often yields an up-scaled profile in seismic.

Most of the method's uncertainties are associated with the inversion procedure itself^45^. First of all, the inversion is nonunique, i.e., several different solutions (combinations of elastic parameters) may yield the same seismic response. Moreover, the need for an initial low-frequency model poses a main uncertainty during the simultaneous AVO inversion. If the low-frequency model is far away from the truth, the inversion cannot predict the correct answer. Since the low-frequency model is generated from the well-log data and seismic velocities, it becomes more uncertain away from the well control affecting offset-to-angle calculations^45^. To verify the predictions of our suggested technique in CO_2_ storage monitoring, saturation calculations from monitoring wells with time-lapse logging can be employed. In case of a hydrocarbon field, comparison with the existing wells (not used for model calibration) can help examine the model-derived saturation accuracy, as in the case of Well-B in Fig. 1. Using this procedure in frontier areas to predict hydrocarbon may be complemented by our proposed method that combines seismic with Controlled Source Electro-Magnetic (CSEM)^39^.

The other uncertainties are the lateral changes in mineralogy or shale volume within the reservoir, resulting in a slight change in the reference brine saturated trend compared to the original calibration. A stochastic approach can be used to address these uncertainties, taking for example, a normal distribution of the input parameters. In the case of two fluids present in a reservoir, i.e., oil with a gas cap are difficult to distinguish; therefore, calibration with gas parameters can be employed to represent the combined influence of the two fluids. A surface draped on an Sw cube may exhibit an 'aliasing pattern' (Fig. 4d–f) depending on the data sampling frequency. The stochastic solution will also resolve this imaging problem.

## Conclusions

The seismic method generally provides the subsurface structural and stratigraphic information. Prestack seismic data can be inverted to provide quantitative information on physical properties such as acoustic impedance (AI), shear impedance (SI), and Vp/Vs ratio. Though seismic velocities are moderately sensitive to the change in saturation, using a combination of AI and Vp/Vs ratio can discriminate fluids and their saturations in many situations.

We introduced a new rock physics model that calculates fluid saturations onto the AI versus Vp/Vs ratio plane directly using the cubes inverted from seismic. Without going into the elastic moduli level and Gassmann substitution, the model can be calibrated using well log data by comparing the S_w_ calculated from AI and Vp/Vs curves with the Archie-derived S_w_. We demonstrated using this model that the elastic properties inverted from seismic help predict CO_2_ saturation in a reservoir during and after injection in a subsurface geological CO_2_ storage.

Modeling using our proposed approach showed that CO_2_ saturation estimation and the plume area delineation is possible using acoustic impedance (AI) and Vp/Vs ratio. The change in pore-pressure estimation is also possible by quantifying the change in brine-sand trend using the stress/cementation coefficient 'n' in un-cemented sand reservoirs. The relation of 'n' with different effective stresses in various uncemented sands warrants further investigation.

One can also use the suggested procedure to monitor oil and gas production and for hydrocarbon exploration. The main uncertainties and pitfalls of the method come from the inherent inversion problems. We expect with the improvement in prestack inversion technology, the predictability of our rock physics model will increase.

## Methods

We generated a rock physics model assuming that a reservoir consists of a rock matrix, pore spaces containing salt water (brine), and other fluids (e.g., CO_2_, or hydrocarbon). According to the assumption, the total volume of rock comprising the matrix and the fluids in the pore spaces is equal to 1. Wyllie^46^ approximated the relation between velocity and volumes in sedimentary rocks with the following expression:

3 $$\frac{1}{{\mathrm{V}}_{\mathrm{P}}}=\frac{(1-\varnothing )}{{\mathrm{V}}_{{\mathrm{P}}_{\mathrm{ma}}}}+\frac{{\mathrm{S}}_{\mathrm{fl}}\varnothing }{{\mathrm{V}}_{{\mathrm{P}}_{\mathrm{fl}}}}+\frac{(1-{\mathrm{S}}_{\mathrm{fl})}\varnothing }{{\mathrm{V}}_{{\mathrm{P}}_{\mathrm{w}}}}$$

where Vp is the P-wave velocities of the saturated rocks, Vp_ma_, Vp_fl_, and Vp_w_ are the P-wave velocities of the rock grains, the pore fluid (other than saltwater), and saltwater (brine), respectively, $\mathrm{\varnothing }$ is the pore space volume. S_fl_ is the target fluid saturation. This equation is often called the time-average equation. It is heuristic and not justifiable theoretically; however, it is useful for estimating P-wave velocity directly without calculating the elastic moduli components. The bulk density (ρ_b_) is a volumetric average of the densities of the rock constituents that can be related to the various rock volume components by:

4 $${\uprho }_{\mathrm{b}}=\left(1-\mathrm{\varnothing }\right){\uprho }_{\mathrm{ma}}+ {S}_{fl}\varnothing {\uprho }_{\mathrm{fl}}+ \left(1-{S}_{fl}\right){\mathrm{\varnothing \rho }}_{\mathrm{w}}$$

where ρ_ma_, ρ_fl_ and ρ_w_ are the densities of rock grains, target fluid, and brine respectively. Combining Eqs. (3), and (4), we obtain an expression in terms of the pore-space volume ($\mathrm{\varnothing }$):

5 $$\varnothing = \frac{\left({\rho }_{ma} - \frac{AI}{ {V}_{{P}_{ma}}}\right)}{\left\{AI\left[{S}_{fl}\left(\frac{1}{{V}_{Pfl}} - \frac{1}{{V}_{Pw}}\right)+\left(\frac{1}{{V}_{Pw}} - \frac{1}{{V}_{Pma}}\right)\right] - \left[{S}_{fl}\left({\rho }_{fl}-{\rho }_{w}\right)+\left({\rho }_{w}-{\rho }_{ma}\right)\right]\right\}}$$

where AI is acoustic impedance. Employing a relation between the S-wave velocity and the P-wave velocity^47^:

6 $$\frac{{V}_{P}}{{V}_{S}}= \frac{1}{\left[G\alpha {\left(1-\varnothing \right)}^{n}\right]}$$

we can calculate the Vp/Vs ratio against a given AI by substituting $\mathrm{\varnothing }$ from Eq. (5). Changing the mineralogy/shaliness coefficient 'G' results in a vertical static shift in the curved iso-saturation lines, α is Vs/Vp ratio of the mineral/rock matrix that defines the matrix-mineral pole on the AI versus Vp/Vs ratio plane. The stress/cementation coefficient 'n' controls the slope of the iso-saturation curved lines and may be selected in a formation zone depending on the level of stress, compaction, or cementation. The relevant constants may be taken from literature^48^ and vendor's logging chart books.

From this function (Eq. 6), we can define a set of lines representing different fluid saturations converging at the 100% matrix-mineral pole on the AI versus Vp/Vs ratio plane (Fig. 7a). Iterating the values of 'G' and 'n' one can calibrate the wet trend of the well data with the 100% S_w_ line (Fig. 7a). Finally, we find out the values of the target fluid's apparent density (ρ_fl_) and apparent P-wave velocity (V_Pfl_) by iterating their values until the S_w_ is computed using Eq. (2) calibrates with the Archie S_w_^49^ (Fig. 7b). The apparent fluid velocity (V_Pfl_) and density (ρ_fl_) values may be fictitious as their difference from the actual values could depend on factors such as the mode of saturation (continuous^50^ or patchy^51^) etc.

The calibrated model then can be applied by inputting the seismic-derived AI and Vp/Vs cubes to obtain an S_w_ cube. A similar approach with different initial assumptions leads to the derivation of a rock physics relation for estimating shale volume (V_sh_) from inverted data^38^.

The original reservoir simulation model was concieved by the Northern Light project^41^. The model simulated one of the potential CO_2_ storage sites “Smeaheia” in the northern North Sea. The injection rate used was 1.3 Mt/year with an injection period of 25 years (from 2020 to 2045). The post-injection period was simulated for 100 years. Subsequently, using results from reservoir simulation, the Norwegian Geotechnical Institute (NGI) generated Vp, Bulk Density and Resistivity^40^ properties. For the present study, we generated Vs data additionally to obtain Vp/Vs ratio cubes by applying Castagna's Eq. ^52^ on the baseline Vp. We assumed that there was no change in shear modulus as the gas injection proceeded, while the change in the density within the plume area was substituted accordingly. Finally, we used the AI (Vp × Bulk Density) and Vp/Vs property cubes in the present study.
